# The ZJU index is associated with the risk of obstructive sleep apnea syndrome in Chinese middle-aged and older people: a cross-sectional study

**DOI:** 10.1186/s12944-023-01974-1

**Published:** 2023-11-29

**Authors:** Liping Wang, Guqiao Nie, Fengqin Yan, Nianli Zhou, Meng Zhang, Wen Peng

**Affiliations:** General Practice Department, Union Hospital, Tongji Medical College, Huazhong University of Science and Technology, 1277 Jiefang Avenue, Jianghan District, Wuhan, China

**Keywords:** ZJU index, Obstructive sleep apnea syndrome, Metabolism

## Abstract

**Background:**

The ZJU index, a novel calculation that combines body mass index, triglycerides, fasting blood glucose and the ratio of alanine aminotransferase to aspartate aminotransferase, is a closely related measure of obesity and insulin resistance. Studies of the ZJU index in relation to obstructive sleep apnea syndrome (OSAS) have not been reported. This study assessed the correlation between the ZJU values and OSAS risk.

**Methods:**

A total of 2,130 participants who underwent polysomnographic monitoring were included in the study. The participants’ basic information and laboratory biochemical indicators were collected, and the ZJU index was computed. The ZJU index was divided into quartiles. The correlation between the different ZJU index levels and OSAS risk was assessed using logistic regression. Drew a receiver operating characteristic (ROC) relationship curve, with prediction efficacy judged by the area under the curve (AUC), and found the optimum cut-off point for ZJU index to predict OSAS. Relative risks were presented as odds ratios (OR). The range of OR values is expressed in the form of 95% confidence intervals (95% CI).

**Results:**

The number of patients diagnosed with OSAS increased progressively with increasing ZJU index (T1: 9.4%; T2: 20.6%; T3: 28.3%; T4: 41.7%; *P* < 0.001). The additional confounders were adjusted by the logistic regression models, the study revealed an independent correlation between ZJU index and OSAS. (*P* < 0.001). The OSAS risk was notably higher at the highest ZJU index levels. (OR = 2.046 [95% CI: 1.057 to 3.964]). The ROC curve for the ZJU index showed an AUC of 0.64 (*P* < 0.001) for males and 0.75 (*P* < 0.001) for females, with a specificity of 64% and 55% and a sensitivity of 60% and 92% for males and females, respectively, with the optimum cut-off values of 36.568 and 34.722, respectively.

**Conclusion:**

A high ZJU index was significantly associated with an increasing risk of OSAS. The ZJU is expected to be a meaningful index for detecting OSAS in the general population.

## Background

It is well acknowledged that obstructive sleep apnea syndrome (OSAS) has become a widespread clinical disease that poses an escalating threat to global public health, particularly amidst the obesity epidemic [1]. Its clinical manifestations mainly include snoring, apnea, and recurrent awakenings. It has also been linked to a decline in cognitive function in some patients, and the symptoms are non-specific; therefore, they are often overlooked [2]. Intermittent hypoxia, hypercapnia promoting inflammation, oxidative stress, and other pathological manifestations of apnea have been reported [3–5]. Among the recognised risk factors for OSAS, age, gender, obesity, inflammation, and disorders of lipid and glucose metabolism are significant contributors to the development of OSAS-related illnesses [6]. This means that OSAS predisposes patients to several secondary metabolic, cardiovascular, and cerebrovascular diseases. OSAS has been shown to be strongly connected with cardiovascular diseases like coronary heart disease and hypertension, [7, 8] metabolic diseases including diabetes mellitus and metabolic syndrome, [9, 10] and cerebrovascular diseases such as dementia and stroke [11, 12]. Currently, the diagnosis of OSAS relies on polysomnographic monitoring and using the apnea-hypopnea index (AHI) to assess severity [13]. Therefore, it is critical to seek simple and easily obtainable biochemical indices to effectively detect OSAS. The ZJU is a new metabolic index that takes into account changes in a number of indicators including aspartate transaminase (AST), alanine aminotransferase (ALT), triglycerides (TG), body mass index (BMI), and blood glucose [14]. The ZJU Index provides a more comprehensive insight and better captures metabolic disorder trends and disease risk than isolated biochemical indicators. Study demonstrates it’s linked to insulin resistance, lipid metabolism and obesity, and it was earliest developed to predict non-alcoholic fatty liver disease (NAFLD) and was validated to have a favourable predictive ability [14, 15]. It has been established that OSAS-related inflammatory mechanisms are closely associated with liver injury in NAFLD [16, 17]. However, there have been no investigations exploring the association between the ZJU index, which includes blood glucose, lipids, and liver function, and the risk of OSAS. The ZJU index can comprehensively reflect the trend of metabolic disorders such as blood glucose and lipids in the earlier stage of a patient’s disease. Consequently, it can be speculated that the ZJU index may be connected to a high risk of OSAS, as well as severity of the disease, and shows promise as a valid predictive tool for OSAS. This can improve the recognition rate of OSAS and facilitate early intervention for the effective prevention of serious complications.

## Methods

### Research object

The analysis included individuals aged 40 years or above, who received medical examinations at the General Department of the Wuhan Union Hospital between December 2019 and January 2022. Only those who underwent polysomnographic monitoring were considered for the study, with a total of 2,998 participants. Participants with any of the following conditions were excluded: persistent severe infection, acute coronary syndrome, stroke, active tumours, dialysis status, multiple organ dysfunction, other acute and serious illnesses; long-term treatment with hormones, antibiotics, or chemotherapeutic agents; and incomplete clinical information. Ultimately, 2,130 individuals were contained within this study. The Institutional Review Board of Tongji Medical College of Huazhong University of Science and Technology approved this study. The Ethics Committee approved the request to waive the requirement for informed consent.

### Clinical characteristics and laboratory measures

All participants underwent routine blood and biochemical measurements and polysomnographic monitoring. General characteristics, such as age, gender, weight, height, smoking and alcohol consumption, as well as the past medical history of the patients, were collected. Combined height and weight to calculated BMI, and according to the Chinese adult BMI classification standard, a BMI equal to or greater than 28 kg/m² was considered obese [18]. Routine blood and biochemical measurements were performed in a fully automated Beckman Coulter LH-750 blood cell analyser (Beckman Coulter Trading Co., Beijing, China) and a Beckman Coulter AU5800 fully automated biochemistry analyser (Beckman Coulter Trading Co., Beijing, China). Venous blood was sampled from patients early the next morning in the fasting state and sent to the central laboratory of the Wuhan Union Medical College Hospital for testing, and all tests were performed by professional laboratory technicians. The tests included white blood cell count, haemoglobin, platelet count, fasting blood glucose (FBG), AST, uric acid, ALT, creatinine, lipid markers such as TG and total cholesterol, and other biochemical markers. The ZJU index was computed by combining BMI with TG, FBG, ALT and AST levels using the following formula : ZJU Index = BMI (kg/m^2^) + FBG (mmol/L) + TG (mmol/L) + 3 × ALT/AST ratio and, in the case of women, adding 2 [14]. The YH-600B sleep apnea monitor (BMC Medical Co., Ltd., Beijing, China) and Polypro S1 1.1.3.0000 sleep analysis software system (BMC Medical Co., Ltd., Beijing, China) were used to record and analyse respiratory events, including apnea, hypoventilation, and other abnormal respiratory events, as well as oxygen saturation and electrocardiographic changes, during the participant’s sleep period throughout the night. According to international diagnostic criteria, apnea is characterized by 90% apnea lasting for a minimum of 10 s, while hypoventilation is defined by events like a 50% decline in airflow and oxygen saturation reduction of 3% for more than 10 s [13]. Sleep monitoring technicians and sleep analysis specialists analysed the respiratory events monitored during sleep and calculated the sleep efficiency, apnea index, oxygen decrement index, hypopnea index, and AHI.

### Statistical analysis

All numerical variables were first tested for normality, and those that were normally distributed were described as means ± SD and analysed using the t-test. Otherwise, they were described as medians (interquartile ranges) and subsequently analysed using the Kruskal-Wallis H-test. The categorical variables were reported in the form of percentages and comparative analyses of differences were performed using the chi-square test. The ZJU index was stratified into quartiles, and the associations between different levels of ZJU values and OSAS risk were assessed using logistic regression models. In addition, the capacity of the ZJU index to forecast OSAS was analysed using receiver operating characteristic (ROC) curves, and the results are reported as an area under the curve (AUC). All data were analysed using SPSS for Windows (version 27.0).

## Results

### General clinical characteristics

A number of 2,130 individuals were involved in this study. Table 1 contains the overall clinical details of the participants. The total population consisted of 68% men and 32% women, and the mean age was 59.84 ± 11.074 years. The prevalence of OSAS was 10.5%. Comparison of the general characteristics in the four subgroups of the ZJU index showed that participants with higher ZJU index levels exhibited higher white blood cell count, total cholesterol, TG, FBG, ALT, and uric acid values, and were at risk of hypertension, diabetes mellitus, obesity, and OSAS (all *P* for trend < 0.001). The number of individuals diagnosed with OSAS increased progressively with increasing ZJU index (T1, 9.4%, T2, 20.6%, T3, 28.3%, T4, 41.7%; *P* < 0.001).

**Table 1:** Basic characteristics of participants in different ZJU index subgroups

Variables	T1(n = 533) ( < 32.40)	T2(n = 532) (32.40-35.02)	T3(n = 534) (35.03–38.19)	T4(n = 531) ( > 38.19)	*P*
Age	61.85 ± 12.35	60.71 ± 10.89	59.04 ± 10.26	57.74 ± 10.26	< 0.001
WBC	4.92 (1.28)	5.22 (1.36)	5.47 (1.83)	5.68 (2.16)	< 0.001
Platelet	194 (72)	201.5 (67.8)	203 (72)	202 (65)	0.004
Neutrophil	2.79 (1.1)	2.91 (1.13)	3.05 (1.49)	3.30 (1.43)	< 0.001
Lymphocyte	1.61 (0.55)	1.75 (0.67)	1.79 (0.64)	1.90 (0.71)	< 0.001
Monocyte	0.34 (0.16)	0.35 (0.18)	0.37 (0.19)	0.38 (0.2)	< 0.001
TG	0.95 (0.53)	1.19 (0.72)	1.44 (1.01)	2.16 (2.22)	< 0.001
TC	4.2 (1.36)	4.23 (1.71)	4.29 (1.64)	4.37 (1.57)	< 0.001
ALT	15 (9)	18 (11)	22 (15)	27 (18)	< 0.001
AST	20 (7)	20 (7)	20 (9)	22 (11)	< 0.001
Creatinine	68 (20.3)	71.3 (20)	70.7 (21.45)	70.1 (15.97)	0.011
Uric acid	313 (125.3)	359.3 (124.4)	366 (130.4)	383.8 (133.4)	< 0.001
FBG	4.69 (0.67)	4.90 (0.74)	5.16 (1.09)	5.68 (1.85)	< 0.001
LDL-C	2.43 (1.23)	2.59 (1.51)	2.55 (1.31)	2.43 (1.33)	0.336
HDL-C	1.36 (0.48)	1.16 (0.46)	1.11 (0.41)	0.97 (0.33)	< 0.001
Sex					< 0.001
Male n (%)	313 (21.6)	370 (25.6)	365 (25.2)	400 (27.6)	
Female n (%)	220 (32.3)	162 (23.8)	169 (24.8)	131 (19.2)	
Tobacco use n (%)	127 (20.2)	159 (25.3)	148 (23.5)	195 (31)	< 0.001
Alcohol use n (%)	109 (18.6)	140 (23.9)	163 (27.8)	175 (29.8)	< 0.001
Hypertension n (%)	205 (16.7)	323 (26.2)	333 (27.1)	370 (30)	< 0.001
Diabetes n (%)	80 (12.7)	128 (20.4)	158 (25.2)	262 (41.7)	< 0.001
OSAS, n (%)	21 (9.4)	46 (20.6)	63 (28.3)	93 (41.7)	< 0.001
CHD, n (%)	84 (23.2)	107 (29.6)	88 (24.3)	83 (22.9)	0.582
Obesity, n (%)	0 (0)	1 (0.3)	33 (11.3)	259 (88.4)	<0.001

Abbreviations: WBC, white blood cell; TG, triglyceride; TC, total cholesterol; ALT, alanine aminotransferase; AST, aspartate aminotransferase; FBG, fasting blood glucose; LDL-C, low-density lipoprotein cholesterol; HDL-C, high-density lipoprotein cholesterol; OSAS, obstructive sleep apnea syndrome; CHD, coronary heart disease

### Associated risk factors for OSAS

A multifactorial logistic regression model was constructed with the risk factors as independent variables and the presence of OSAS as the dependent variable. Variables such as smoking, alcohol consumption, diabetes mellitus, obesity, leukocytes, haemoglobin, platelets, neutrophils, monocytes, lymphocytes, ALT, creatinine, uric acid, AST, TG, and total cholesterol, etc. were taken into account in the model, while the ZJU index was considered a continuous variable. The results obtained after adjusting for confounders are presented in Table 2. Sex, hypertension, monocytes and ZJU index remained significantly positively associated with the OSAS risk after adjusting for confounders. Possibly due to the limitations in the sample size of the study population, this study did not find a positive association between age and the risk of developing OSAS.

**Table 2:** Multifactorial regression analysis of OSAS-related risk factors

Variables	OR	95% CI	*P*
Monocyte	3.814	1.032–14.09	0.045
Sex	2.529	1.533–4.172	< 0.001
Hypertension	1.618	1.138-2.300	0.007
Neutrophil	1.374	0.739–2.552	0.315
LDL- C	1.298	0.899–1.875	0.164
Lymphocyte	1.297	0.645–2.607	0.466
Diabetes	1.253	0.880–1.785	0.212
Tobacco use	1.212	0.842–1.745	0.301
Obesity	1.171	0.702–1.954	0.545
ZJU index	1.117	1.041–1.198	0.002
Alcohol use	1.077	0.746–1.553	0.694
TG	1.011	0.886–1.154	0.869
Haemoglobin	1.003	0.994–1.013	0.491
Platelet	1.001	0.998–1.004	0.465
Creatinine	1.000	0.996–1.004	0.860
Uric acid	1.000	0.999–1.001	0.786
ALT	0.996	0.983–1.009	0.556
AST	0.994	0.973–1.015	0.548
Age	0.973	0.956–0.990	0.002
FBG	0.901	0.796–1.019	0.096
HDL- C	0.869	0.646–1.167	0.350
TC	0.752	0.557–1.014	0.062
WBC	0.704	0.381–1.302	0.263

Abbreviations: LDL-C, low-density lipoprotein cholesterol; TG, triglyceride; ALT, alanine aminotransferase; AST, aspartate aminotransferase; FBG, fasting blood glucose; HDL-C, high-density lipoprotein cholesterol; TC, total cholesterol; WBC, white blood cell

### Logistic regression analysis among ZJU index values and OSAS

The ZJU index values were stratified using the quartile method. The relationship of ZJU levels with the risk of OSAS was evaluated using logistic regression. The results are summarised in Table 3. Compared to T1 as the reference level, the analysis revealed a significant increase in the risk of OSAS with the ZJU index at T4 (OR = 2.851, 95% CI: 1.629–4.988, *P* < 0.001), as shown in Model 2. The risk decreased slightly after adjusting for the remaining covariates in Model 3; however, it persisted (OR = 2.046, 95% CI: 1.057–3.964, *P* = 0.034). The risk of OSAS progressively increased with rising ZJU index (T3 compared to T1, OR = 2.109, 95% CI: 1.218–3.652; T4 compared to T1, OR = 2.046, 95% CI: 1.057–3.964).

**Table 3:** Logistic regression analysis among ZJU index values and OSAS

ZJU index	OR (95% CI)	*P*	OR (95% CI)	*P*	OR (95% CI)	*P*
	Model 1		Model 2		Model 3	
T1	-		-		-	
T2	2.308 (1.357–3.924)	0.002	2.002 (1.171–3.423)	0.011	1.647 (0.948–2.862)	0.076
T3	3.261 (1.959–5.428)	<0.001	2.63 (1.564–4.422)	<0.001	2.109 (1.218–3.652)	0.008
T4	5.177 (3.170–8.454)	<0.001	2.851 (1.629–4.988)	<0.001	2.046 (1.057–3.964)	0.034

Model 1: crude model

Model 2: adjusted for age, sex, smoking, alcohol, and obesity

Model 3: adjusted for age, sex, smoking, alcohol, obesity, hypertension, diabetes, fasting blood glucose, white blood cell, haemoglobin, platelets, monocytes, neutrophils, lymphocytes, creatinine, uric acid, alanine aminotransferase, total cholesterol, high-density lipoprotein cholesterol, low-density lipoprotein cholesterol

### Correlation analysis between ZJU index and abnormal respiratory index

**Fig. 1:**
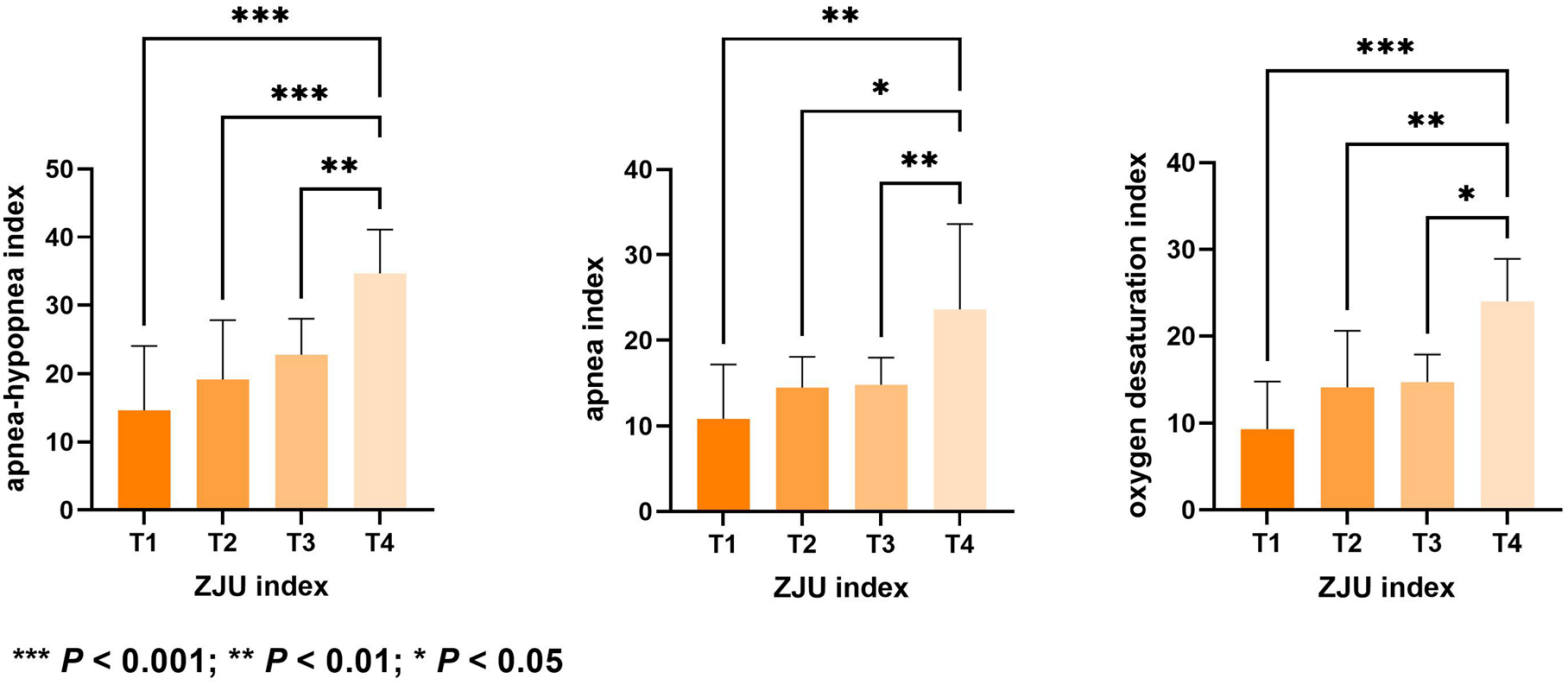
Comparison of abnormal respiration-related index values in different ZJU index groups

Table 4 shows the relationship between ZJU and the abnormal respiratory indices in patients with OSAS. The ZJU index exhibited significant positive correlations with the AHI, apnea index, and oxygen desaturation index. According to Fig. 1, the AHI, oxygen desaturation index, and apnea index values were notably greater in the T4 group with high ZJU values compared to the T3, T2, and T1 groups (All *P*-values < 0.05).The AHI reflects the severity of OSAS, [13] therefore, it can be surmised that the ZJU index may correlate with the severity of OSAS.

**Table 4:** Correlation analysis between ZJU index and abnormal respiratory index

Variables	r	*P*	95% CI
apnea-hypopnea index	0.259	< 0.001	0.126–0.383
oxygen desaturation index	0.231	0.001	0.096–0.357
apnea index	0.207	0.003	0.071–0.335
hypopnea index	0.105	0.137	-0.033-0.239
sleep efficiency	-0.02	0.773	-0.158-0.118

### ROC curve

**Fig. 2:**
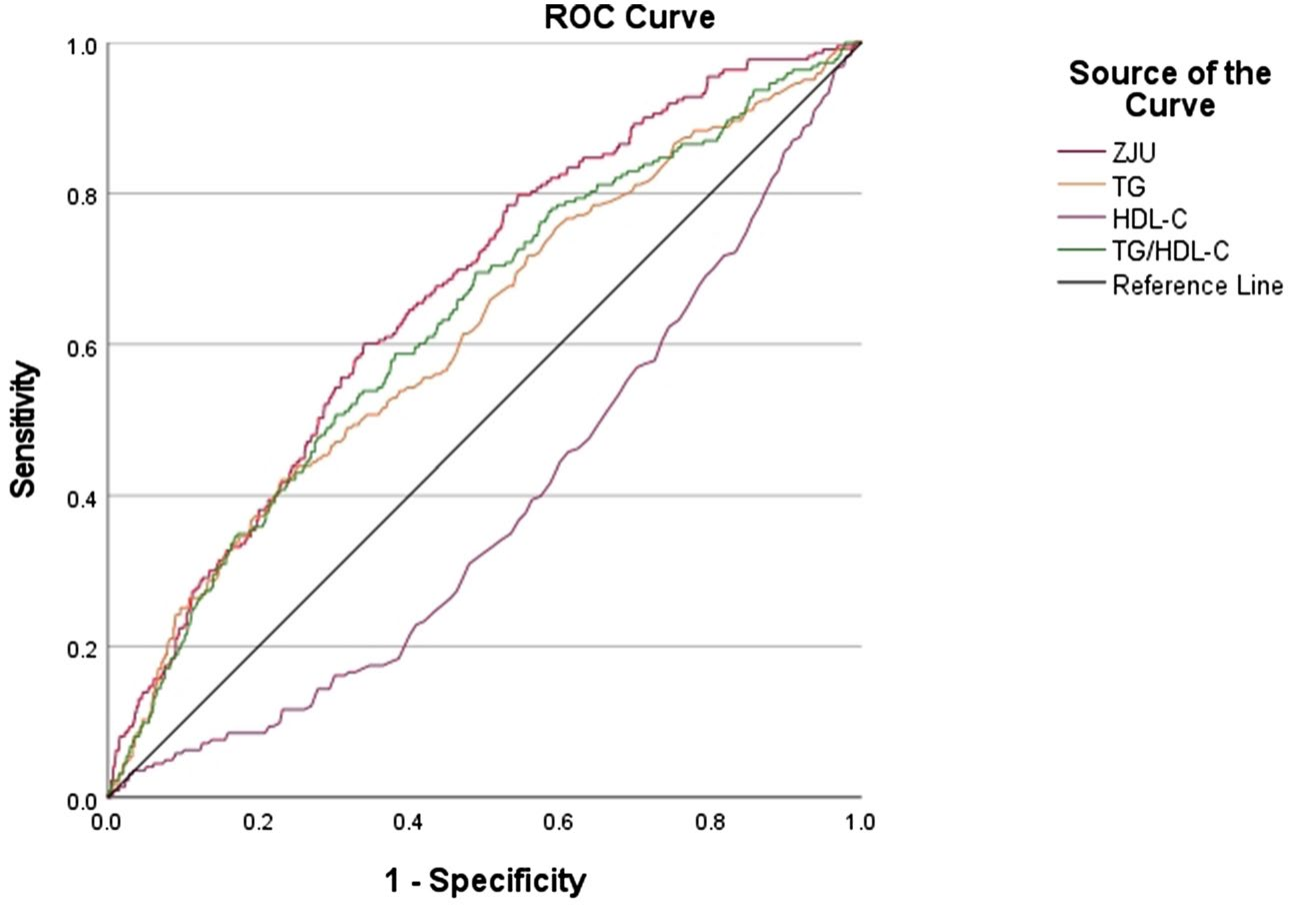
Identification of ROC curves for OSAS using various lipid markers

Lipid metabolism indicators associated with OSAS, such as TG, High-density lipoprotein cholesterol (HDL-C), the ratio of TG/HDL-C, and the ZJU index, were screened using univariate logistic regression, and the ROC curves of these indicators versus OSAS were drawn. Figure 2 illustrates these results. The areas under the ROC curve, ranked in descending order, were as follows: the ZJU index (AUC = 0.667), the ratio of TG/HDL-C (AUC = 0.630), TG (AUC = 0.615), and HDL-C (AUC = 0.386), all *P*-values < 0.001. This indicates that the ZJU index is more effective in identifying individuals who are at high probability of having OSAS.

**Fig. 3:**
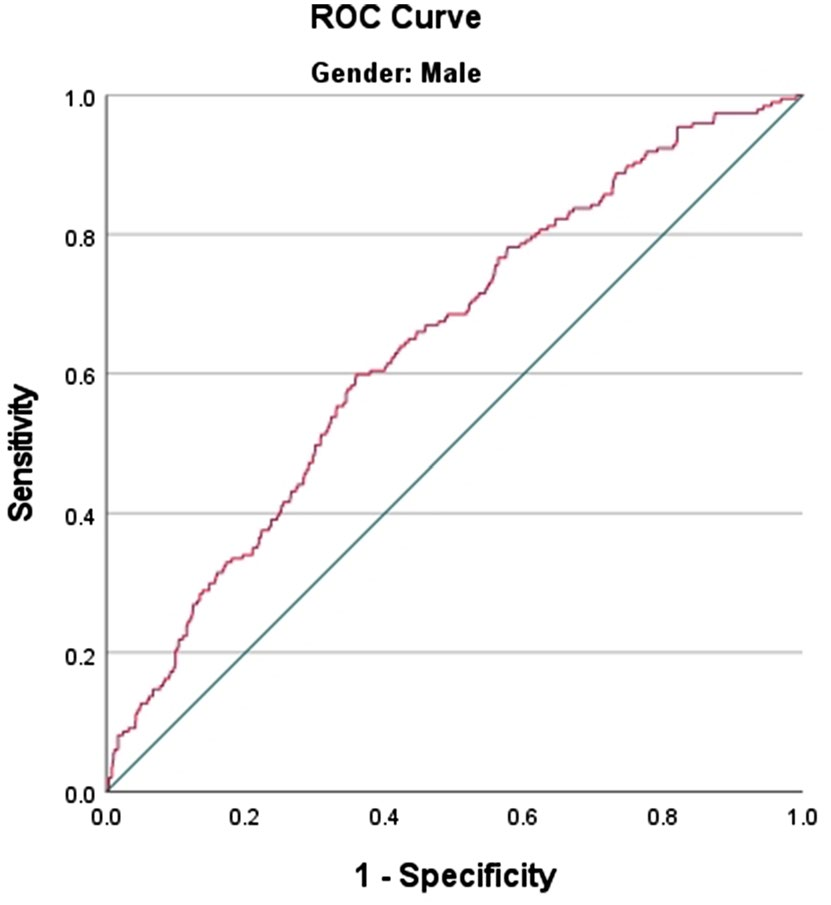
Identification of ROC curves in male OSAS using the ZJU index

**Fig. 4:**
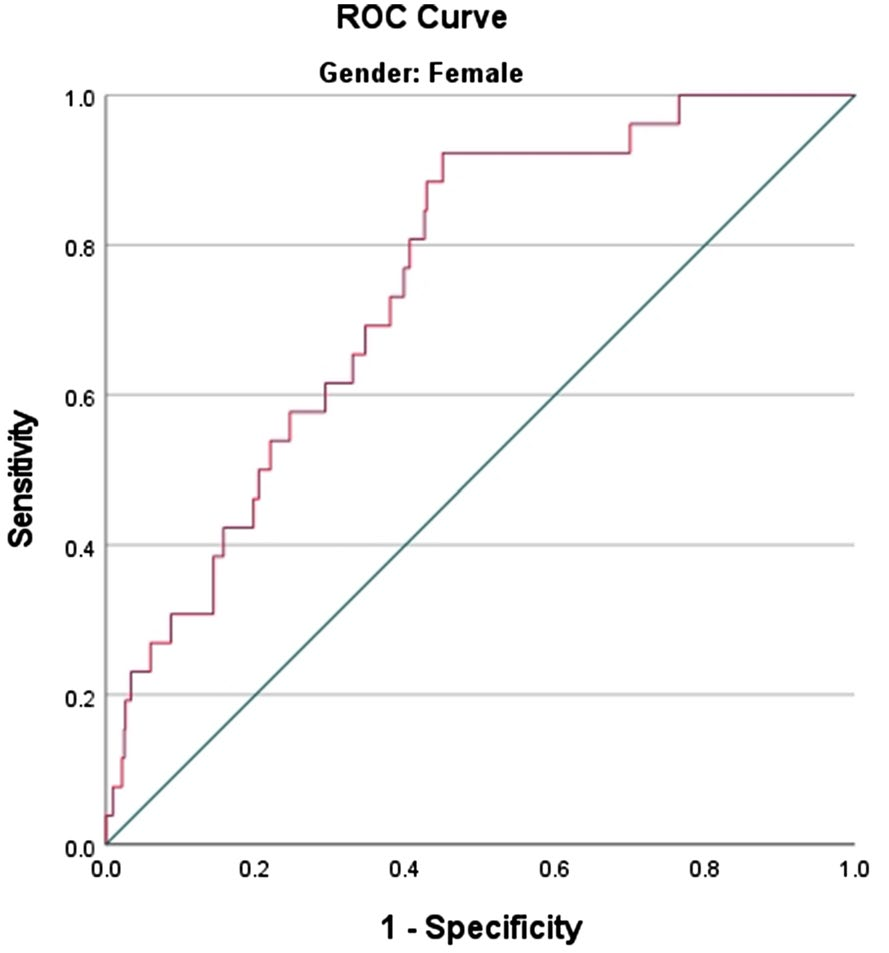
Identification of ROC curves in female OSAS using the ZJU index

The ROC curves of the ZJU index versus OSAS for different sexes are plotted in Figs. 3 and 4. This study found that the AUC value for males was 0.641 (*P* < 0.001), whereas the AUC value for females was 0.750 (*P* < 0.001). The sensitivity and specificity were 60% and 92% and 64% and 55%, respectively. The optimal cut-off values were 36.568 and 34.722, respectively. The AUC value was higher for females than for males, with a sensitivity of 92%. This finding suggests that the ZJU index is more effective in screening high-risk women with OSAS. This is due to the fact that superior screening is achieved through high sensitivity.

## Discussion

OSAS is a common respiratory condition that has become more prevalent due to the rise of obesity [19]. Intermittent hypoxia associated with OSAS is also associated with the onset and progression of various other diseases [20]. Moreover, untreated OSAS carries the possible risk of serious complications and mortality [21, 22]. Oxidative stress caused by hypoxia affects cellular metabolism and leads to endothelial cell dysfunction, which are the main pathological mechanisms underlying the progression of cardiovascular and cerebrovascular disease in patients with OSAS [23]. Studies have shown that OSAS severity correlates with microangiopathy [24]. A prospective study conducted within the community also validated the correlation between OSAS and an increased likelihood of atrial fibrillation [25]. Early screening of individuals at high risk of OSAS and interventions to reduce risk factors are critical. The diagnosis of OSAS still relies on polysomnography as the preferred method. Nevertheless, the practical use of it is limited due to the duration and expense of the examination, along with the requirements for patient adherence and collaboration. Hence, it is necessary to discover affordable, convenient, and efficient biomarkers for early detection of metabolic disorders and forecasting the likelihood of OSAS.

Various studies have established that higher cholesterol and blood glucose levels are independently connected to OSAS disease [26–28]. Several genes involved in cholesterol metabolism are affected in patients with OSAS, which can lead to inflammation and adipose tissue dysfunction [29, 30]. Studies on chronic liver injury in OSAS have revealed that OSAS is an independent factor that increases the risk of liver enzyme irregularities, [31] which may be caused by insulin resistance, [32] and that inflammation due to hypoxia is also a possible cause of liver dysfunction [33]. The ZJU index, initially created to detect NAFLD in Chinese adults, which is a combination of BMI and FBG, TG, ALT, and AST values [14]. Following that, cohort studies have shown the strong predictive capability of the ZJU index in identifying NAFLD among overweight women in Western countries [34]. It is tightly related to obesity and metabolic disorders and is a useful surrogate indicator of insulin resistance [35]. These risk factors are closely related to OSAS; nevertheless, no study has investigated the connection between the level of the ZJU index and OSAS.

The research discovered a strong correlation between the ZJU index and OSAS, indicating that the likelihood of developing OSAS rises as the ZJU index values increase. If the ZJU index exceeded 36.568 for men and 34.722 for women, the patient was at a high risk of developing OSAS. ZJU index has a higher identification power and better screening ability in women. Hence, it is advantageous to identify individuals who are at risk of OSAS at an early stage by assessing the ZJU index value, as this facilitates the implementation of preventive measures and treatment before the onset of the disease. The results of the current research strongly support the clinical use of ZJU index values for early screening of individuals with a high likelihood of developing OSAS disease, and also advise patients to strictly control their blood glucose, lipids and body weight within the normal range through exercise and dietary control, in order to promptly control metabolic disorders and promote a healthy metabolic state, improve health problems. To validate the significance of the ZJU index in diagnosing OSAS, it is recommended to carry out future multicentre cohort studies with larger sample sizes. This is because these tests are usually more readily available than polysomnography. Disorders of glucose and lipid metabolism and hypoxia-induced inflammation are important pathological processes in OSAS, and the associated molecular mechanisms remain unclear and require further study.

## Advantages and limitation

The present research is the initial study to demonstrate an association between the ZJU value and the likelihood of developing OSAS, validating the ability of the ZJU value to forecast the OSAS risk. Furthermore, the findings of this research demonstrated a notable correlation that varies with the dosage between the ZJU index and the likelihood of OSAS. Specifically, a higher ZJU index was found to be linked to a higher risk of developing OSAS, even though confounding factors were taken into account. It also exhibited that the ZJU index showed a positive trend of correlation with indicators reflecting the severity of OSAS disease.

Nevertheless, there are certain constraints that should be taken into account in relation to this research. Firstly, this was a transversal observational study and did not reflect whether there was a causal connection between the ZJU index and OSAS. Secondly, it was a small-sample, single-centre study conducted in the general population, and the conclusions have limited applicability. Finally, the study did not collect variables that included participants’ diet and exercise habits, which could potentially affect the levels of biochemical indicators such as lipids and blood glucose.

## Conclusion

This study indicated that there was a notable correlation between the ZJU index and the likelihood of OSAS, with a higher ZJU level being associated with a greater risk of developing OSAS. It was also closely related to the severity of OSAS. The ZJU index is an appropriate indicator to identify individuals with OSAS in the general population, and its ability to discriminate is stronger in women compared to men. The findings of this study support clinicians in screening and identifying patients with OSAS who require management and follow-up by monitoring the ZJU index in their clinical practice. Additionally, the risk of OSAS can be reduced by promoting patient self-management through health education and appropriate nursing interventions to enable patients to control their weight, fasting blood glucose, blood lipids, and liver function within the normal ranges.

## Data Availability

The datasets used and analysed in the current study are available from the corresponding author upon reasonable request.

## List of abbreviations

OSAS: obstructive sleep apnea syndrome
AHI: apnea-hypopnea index
BMI: body mass index
FBG: fasting blood glucose
TG: triglyceride
AST: aspartate transaminase
ALT: alanine aminotransferase
NAFLD: non-alcoholic fatty liver disease
HDL-C: high-density lipoprotein cholesterol
ROC: receiver operating characteristic
AUC: area under the curve
OR: odds ratio
95% CI: 95% confidence interval
